# Gender and geographic representation in editorial boards of education journals

**DOI:** 10.3389/fpsyg.2024.1330316

**Published:** 2024-02-07

**Authors:** Yan Xue, Quan Xu

**Affiliations:** ^1^Faculty of Artificial Intelligence in Education, Central China Normal University, Wuhan, China; ^2^School of Foreign Languages, Central China Normal University, Wuhan, China

**Keywords:** gender diversity, geographical diversity, editorial board, education, leadership

## Abstract

**Background:**

Previous studies have examined the gender and geographic diversity within editorial boards across various academic disciplines, excluding the field of education. Thus, the purpose of this study was to address this gap by investigating the extent of gender and geographic disparity within the editorial boards of education journals.

**Methods:**

The selection of top five education journals from each quartile (Q1–Q4) was performed based on Clarivate Analytics’ Journal Citation Reports (JCR) 2021 within the category of “Education & Educational Research.” The information of editors was collected through online sources.

**Results:**

Overall, female editors accounted for 17 out of the 29 editors-in-chief (58.62%), 36 out of the 64 deputy/associate editors (56.25%), 378 out of the 728 editorial/advisory board members (51.92%) and 15 out of the 28 other types of editors (53.57%). There was no significant association between the impact factor (IF) and the proportion of female editors (Pearson’s *r* = −0.095, *p* = 0.689). The United States had the highest number of editors (*n* = 459, 54.06%), followed by the United Kingdom (*n* = 98, 11.54%), Australia (*n* = 63, 7.42%), China (*n* = 29, 3.42%), Germany (*n* = 25, 2.94%), and Canada (*n* = 22, 2.59%). Notably, the majority of the included editors were from developed countries (*n* = 794, 93.52%), while editors from developing countries constituted a significantly smaller proportion (*n* = 55, 6.48%).

**Conclusion:**

Editorial boards of education journals exhibit reasonable gender diversity than other disciplines, though still fall short when considering the proportion of women in the discipline. Besides, obvious geographical disparity was observed among editorial boards of education journals. There was a notable lack of representation of researchers associated with institutions from developing countries on the editorial boards of education journals. While maintaining sufficient gender diversity, it is imperative to enhance the geographical diversity in these journals, ensuring a more equitable number of positions to individuals from these underrepresented groups.

## Introduction

In recent decades, there has been a notable increase in the proportion of women opting for faculty positions (Ingersoll et al., 2021). Previous investigation performed by the Association of American Medical Colleges (AAMC) has showed a consistent upward trend in the representation of full-time female faculty in academic medicine since 2009, with women now constituting approximately half of all faculty (AAMC, 2022). Despite this encouraging growth in female participation within the educational community, gender inequities persist among faculty (Casad et al., 2022). It is critical to recognize that gender diversity plays a pivotal role in enhancing academic excellence (Jagsi et al., 2020). According to a comprehensive nationwide survey conducted on 95 sociology departments, it has been observed that women face a 29% lower likelihood of attaining tenure compared to their male counterparts, and additionally experience a prolonged duration in achieving this academic milestone (Weisshaar, 2017). Women comprised only 28% of full professors and 22% of department chairs and permanent medical school deans (Starchl et al., 2023). This underrepresentation of women in the STEM tenure-track pipeline can be attributed to a disparity in the likelihood of women Ph.D. holders applying for tenure-track positions compared to their male counterparts, rather than women facing higher rates of rejection (Ceci et al., 2014). The academic development of women is influenced by various factors, including the absence of role recognition, familial obligations, and cultural bias (Zhuge et al., 2011). The “Glass ceiling,” a term associated with the phenomenon where women face lower odds of advancing to higher positions in organizational hierarchies compared to men, has garnered increased attention in recent years (Horrocks, 2019). Efforts have been made to analyze gender-based trends and determine if women can overcome the invisible barriers hindering their career growth (Rodríguez et al., 2022). The global and hierarchical disparities in the female representation in scientific fields contribute to the observation that men tend to publish, collaborate, and receive more citations than their female counterparts. The gender disparity in academic publishing is evident, as men tend to have a significantly higher publication rate compared to women (Larivière et al., 2013). This discrepancy has been observed to decrease over a span of less than 10 years, with men publishing an average of 13.2 articles, while their female counterparts publish 9.6 articles throughout their careers (Huang et al., 2020). Additionally, men are found to be 30% more likely to receive citations compared to women (Huang et al., 2020). Moreover, previous research has highlighted the underrepresentation of women in senior author positions, which undoubtedly hinders progress toward achieving gender balance in higher academic positions (Pinho-Gomes et al., 2020). These findings raise concerns regarding potential biases that females may face in the scholarly publication, emphasizing the need for addressing these issues (Silver, 2019). Editorial board membership is widely regarded as an indicator of authority and distinction within the realm of academic research (Doja et al., 2014). Journal editors assume a crucial role as guardians of scientific knowledge, shaping policies, steering scientific developments, and ultimately impacting the professional progress of individuals who subsequently shape academic and pedagogical initiatives (Lin and Li, 2023). Recently, there has been a growing emphasis on promoting diversity in editorial leadership, particularly with regards to gender and geographic representation. The lack of gender diversity of editor boards may signify a dearth of equitable opportunities, potentially impeding peer acknowledgement (Gallivan et al., 2021).

The disparity in gender diversity within editorial boards has a consequential impact on the proportion of senior authors, thereby exacerbating concerns regarding female representation (Last et al., 2022). Numerous studies have been conducted to examine the gender composition of editors across various disciplines, revealing a disconcerting prevalence of women’s underrepresentation on editorial boards (Kennedy et al., 2001; Jagsi et al., 2020; Pinho-Gomes et al., 2021). Nevertheless, it is heartening to note that acknowledging this dearth of gender diversity can effectively enhance female representation (Jagsi et al., 2020). Education is a significant interdisciplinary domain that has had a profound impact on various fields, including medicine, psychology, sociology, and economics (Osborne and Mollette, 2010; Lovakov and Yudkevich, 2023). According to research conducted by the United Nations Educational, Scientific and Cultural Organization (UNESCO), the proportion of female teachers in different levels of education varied in 2000, with 92% in pre-primary, 60% in primary, 54% in lower secondary, 48% in upper secondary, and 39% in tertiary education. Investigation in 2021 indicated that these proportions had increased to 94, 67, 58, 51, and 43%, respectively, (UNESCO, 2022). The increasing presence of female teachers has led to a heightened focus on their professional advancement. Addressing and overcoming obstacles that hinder female involvement in educational research and subsequent publication is a crucial matter requiring attention. However, the issue of gender diversity within the editorial boards of education journals is often overlooked. To assess the extent of gender disparity in educational research and publication, this study aims to examine the representation of gender and geographical region within the editorial boards of education journals, which is expected to provide strategies to improve equity and opportunity for women editors.

## Methods

This cross-sectional study examined the gender representation and geographical among the editorial members of education journals deemed representative. As the study relied on publicly accessible data, the institutional review board of Central China Normal University waived the need for ethical approval and informed consent.

The selection of education journals was based on “Clarivate Analytics” Journal Citation Reports (JCR) 2021, within the category of “Education and Educational Research.” For this study, the top five journals from each quartile (Q1–Q4) of the category of ‘Education & Educational Research’ were chosen based on their impact factor (González-Alvarez and Cervera-Crespo, 2019; González-Alvarez and Sos-Peña, 2020). The study acquired the editorial team memberships from the websites of the respective journals, and we extracted the names, geographical location based on place of work, editorial board roles and affiliations of editors. As shown in Table 1, the included editors are divided into four categories according to their positions. Honorary Editor-in-Chief, ethic editors, corresponding editors, biostatistics editors, manuscript editors, language editors, editorial assistants, etc., were not included in this analysis. Gender of editors was determined by examining their profiles or pronouns showed on the home page of journals or on their affiliated websites, Google search engine and so on (Pinho-Gomes et al., 2021; McMullen et al., 2022).

**Table 1:** Gender ratio of editors among 20 journals in the education category.

Journal	Impact factor	Quartile	Editor-in-chief		Deputy/Associate editors		Editorial/Advisory board members		Other editors		The total proportion of women
			Male	Female	Male	Female	Male	Female	Male	Female	
Review of Educational Research	13.551	Q1	–	100.00% (*n* = 4)	50.00% (*n* = 4)	50.00% (*n* = 4)	38.89% (*n* = 28)	61.11% (*n* = 44)	–	–	61.90% (*n* = 52)
Computers & Education	11.182	Q1	100.00% (*n* = 1)	–	–	100.00% (*n* = 1)	65.62% (*n* = 21)	34.38% (*n* = 11)	50.00% (*n* = 2)	50.00% (*n* = 2)	36.84% (*n* = 14)
Educational Research Review	10.207	Q1	100% (*n* = 1)	–	66.67% (*n* = 2)	33.33% (*n* = 1)	57.14% (*n* = 28)	42.86% (*n* = 21)	–	–	41.51% (*n* = 22)
Internet and Higher Education	8.591	Q1	50.00% (*n* = 1)	50.00% (*n* = 1)	100.00% (*n* = 1)	–	44.44% (*n* = 12)	55.56% (*n* = 15)	–	100.00% (*n* = 1)	54.84% (*n* = 17)
Educational Psychologist	8.209	Q1	50.00% (*n* = 1)	50.00% (*n* = 1)	–	–	53.85% (*n* = 28)	46.15% (*n* = 24)	–	–	46.30% (*n* = 25)
Race Ethnicity and Education	3.514	Q2	100.00% (*n* = 1)	–	50.00% (*n* = 3)	50.00% (*n* = 3)	43.24% (*n* = 32)	56.76% (*n* = 42)	–	–	55.56% (*n* = 45)
Educational Assessment Evaluation and Accountability	3.479	Q2	50.00% (*n* = 1)	50.00% (*n* = 1)	–	–	55.17% (*n* = 16)	44.83% (*n* = 13)	–	–	45.16% (*n* = 14)
AERA Open	3.427	Q2	–	100.00% (*n* = 1)	33.33% (*n* = 5)	66.67% (*n* = 10)	31.25% (*n* = 15)	68.75% (*n* = 33)	–	100.00% (*n* = 5)	71.01% (*n* = 49)
Journal of Science Education and Technology	3.419	Q2	100.00% (*n* = 1)	–	–	–	52.27% (*n* = 23)	47.73% (*n* = 21)	–	–	46.67% (*n* = 21)
TESOL Quarterly	3.410	Q2	50.00% (*n* = 1)	50.00% (*n* = 1)	–	–	61.11% (*n* = 22)	38.89% (*n* = 14)	50.00% (*n* = 4)	50.00% (*n* = 4)	41.30% (*n* = 19)
Journal of Educational Change	2.418	Q3	–	100.00% (*n* = 1)	–	–	47.83% (*n* = 11)	52.17% (*n* = 12)	–	–	54.17% (*n* = 13)
Australian Journal of Education	2.415	Q3	–	100.00% (*n* = 1)	–	100.00% (*n* = 3)	71.43% (*n* = 10)	28.57% (*n* = 4)	–	–	44.44% (*n* = 8)
Journal of American College Health	2.394	Q3	–	100.00% (*n* = 1)	75.00% (*n* = 3)	25.00% (*n* = 1)	47.83% (*n* = 11)	52.17% (*n* = 12)	–	–	50.00% (*n* = 14)
Academic Psychiatry	2.385	Q3	100.00% (*n* = 1)	–	75.00% (*n* = 6)	25.00% (*n* = 2)	50.00% (*n* = 9)	50.00% (*n* = 9)	–	–	40.74% (*n* = 11)
Australian Educational Researcher	2.383	Q3	100.00% (*n* = 1)	–	28.57% (*n* = 2)	71.43% (*n* = 5)	28.57% (*n* = 6)	71.43% (*n* = 15)	–	–	68.97% (*n* = 20)
Journal of Beliefs & Values-Studies in Religion & Education	1.724	Q4	100.00% (*n* = 1)	–	50.00% (*n* = 1)	50.00% (*n* = 1)	70.27% (*n* = 26)	29.73% (*n* = 11)	50.00% (*n* = 1)	50.00% (*n* = 1)	30.95% (*n* = 13)
European Educational Research Journal	1.701	Q4	50.00% (*n* = 1)	50.00% (*n* = 1)	–	–	65.52% (*n* = 19)	34.48% (*n* = 10)	75.00% (*n* = 3)	25.00% (*n* = 1)	34.29% (*n* = 12)
Research in Science & Technological Education	1.697	Q4	–	100.00% (*n* = 2)	100.00% (*n* = 1)	–	54.29% (*n* = 19)	45.71% (*n* = 16)	–	–	47.37% (*n* = 18)
Journal of Educational Research	1.670	Q4	–	100.00% (*n* = 1)	–	–	26.09% (*n* = 6)	73.91% (*n* = 17)	75.00% (*n* = 3)	25.00% (*n* = 1)	67.86% (*n* = 19)
Early Childhood Education Journal	1.656	Q4	–	100.00% (*n* = 1)	–	100.00% (*n* = 5)	19.05% (*n* = 8)	80.95% (*n* = 34)	–	–	83.33% (*n* = 40)
Total proportion			41.38% (*n* = 12)	58.62% (*n* = 17)	43.75% (*n* = 28)	56.25% (*n* = 36)	48.08% (*n* = 350)	51.92% (*n* = 378)	46.43% (*n* = 13)	53.57% (*n* = 15)	52.53% (*n* = 446)

Top five education journals from each quartile (Q1–Q4) are listed in order of impact factor. –, Not available.

## Results

Data was collected on a total of 853 editors from 20 educational journals, with 4 editors excluded due to indeterminate gender information. In aggregate, female editors comprised 446 out of the 849 editors (52.53%). When examining specific positions, female editors accounted for 17 out of the 29 editors-in-chief (58.62%), 36 out of the 64 deputy/associate editors (56.25%), 378 out of the 728 editorial/advisory board members (51.92%) and 15 out of the 28 other types of editors (53.57%).

**Figure 1:**
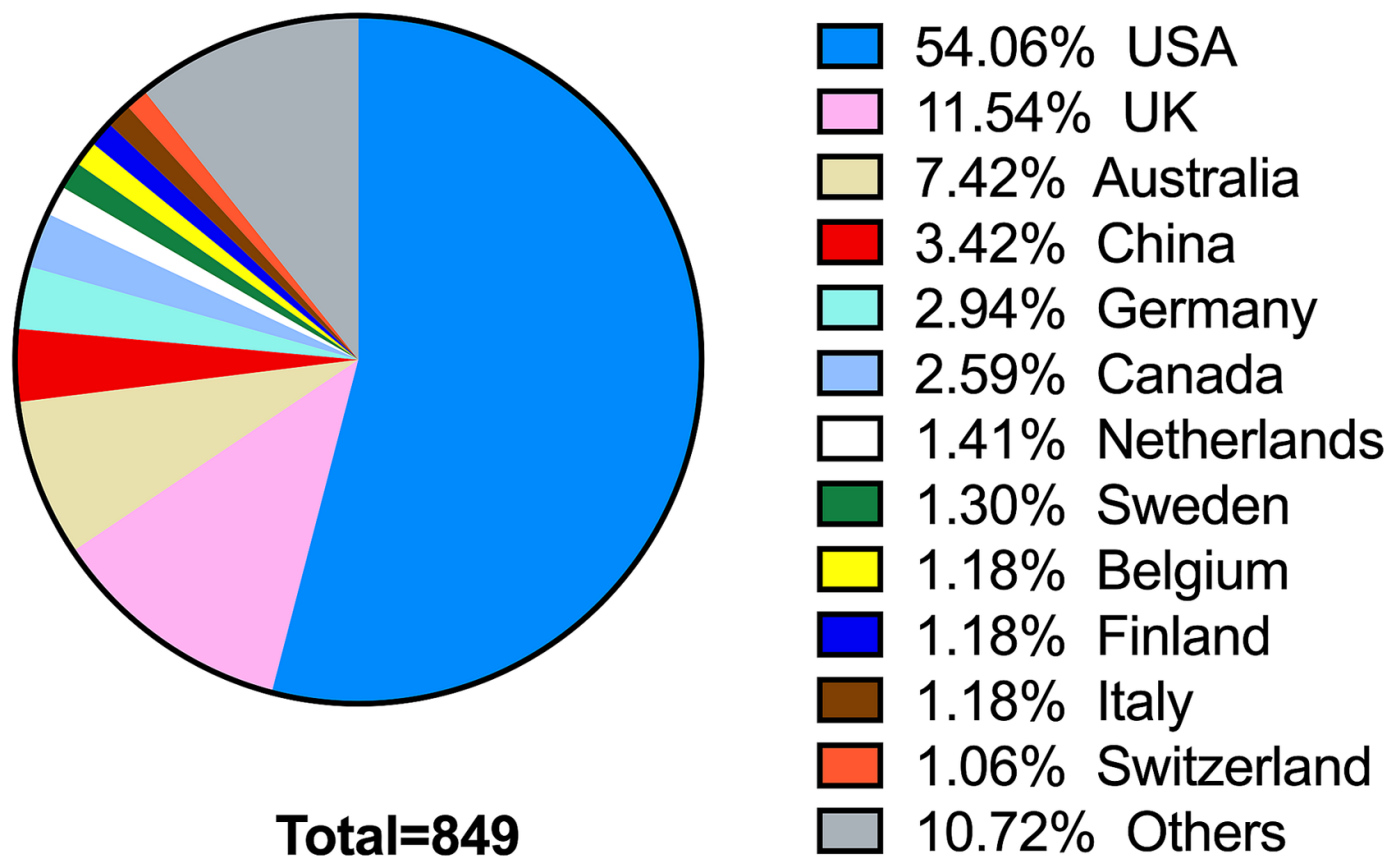
Country distribution of editors of education journals. For ease of presentation, only the countries with percentage above 1% were listed.

Furthermore, according to the correlation analysis, the impact factor was not significantly correlated with the proportion of female editors (Pearson’s *r* = −0.095, *p* = 0.689). The graphical representation in Figure 1 demonstrated the geographical distribution of the editors which based on their work place, with a total of 42 countries represented. Among these, the United States had the highest number of editors (*n* = 459, 54.06%), followed by the United Kingdom (*n* = 98, 11.54%), Australia (*n* = 63, 7.42%), China (*n* = 29, 3.42%), Germany (*n* = 25, 2.94%), and Canada (*n* = 22, 2.59%). Notably, the majority of the included editors were from developed countries (*n* = 794, 93.52%), while editors from developing countries constituted a significantly smaller proportion (*n* = 55, 6.48%).

**Figure 2:**
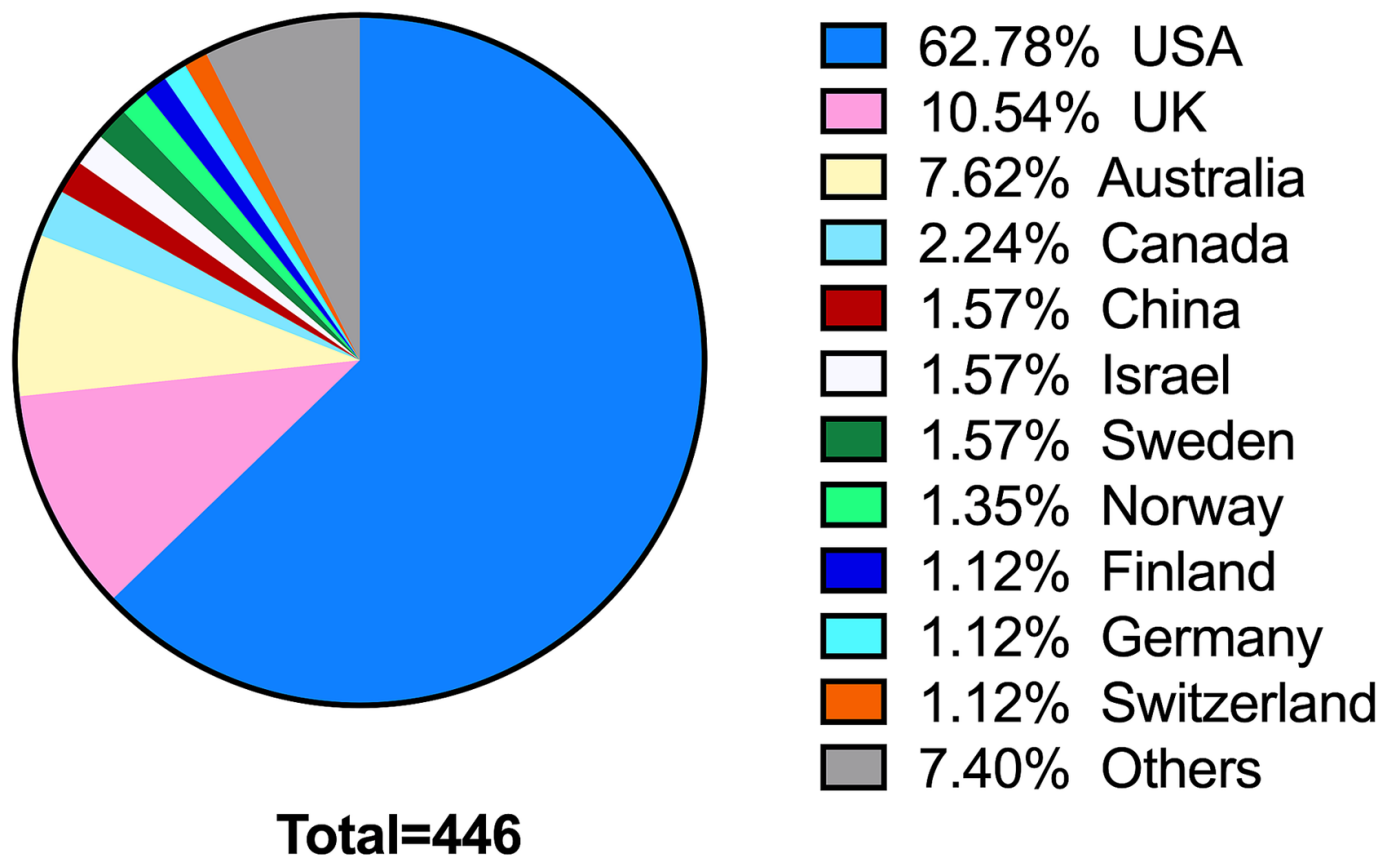
Country distribution of female editors of education journals. For ease of presentation, only the countries with percentage above 1% were listed.

Additionally, as illustrated in Figure 2, the female editors included in the study were sourced from a total of 30 countries, with the United States representing the largest proportion (*n* = 280, 62.78%), followed by the United Kingdom (*n* = 47, 10.54%), Australia (*n* = 34, 7.62%), Canada (*n* = 10, 2.24%), China (*n* = 7, 1.57%), Israel (*n* = 7, 1.57%), and Sweden (*n* = 7, 1.57%). Furthermore, it is noteworthy that the majority of these female editors (96.64%, *n* = 431) hailed from developed nations, while a mere 3.36% (*n* = 15) were from developing countries.

## Discussion

To our knowledge, this study represents the initial endeavor to comprehensively examine the gender and geographic diversity within the editorial boards of education journals. Our investigation successfully discerned the extent of women’s involvement in the editorial boards of the 20 selected educational journals, thereby offering an initial basis for enhancing diversity within such journals.

It is irrefutable that women remain inadequately represented across diverse scientific domains, a disparity that further exacerbates with the rise in impact factor (Bendels et al., 2018; Liévano-Latorre et al., 2020). Several investigations demonstrated this disparity: 28.7% in Biology Conservation (Liévano-Latorre et al., 2020), 19% of women in Business and Management (Metz et al., 2016), 33% in Psychology (Huang et al., 2020), 30.4% in Psychiatry (Hafeez et al., 2019), 24% in Oncology (Dai et al., 2022), 20% in Geology (Henriques and Garcia, 2022), 27.3% in infectious disease and microbiology (Ayada et al., 2022), and 23% in environmental sciences (Lobo-Moreira et al., 2023). Encouraging equity is beneficial to build a culture of equivalence for professionals and their careers (Alkhawtani et al., 2021). Our findings showed that nearly half of editors in education journals are women, which is a higher proportion than in other disciplines. However, when considering the gender diversity within disciplines, it is imperative to examine the representation of women within those disciplines. It is noteworthy that the investigation from Organization for Economic Co-operation and Development showed that approximately 70% of teachers globally are women, indicating a significant proportion (OECD iLibrary, 2019). Compared to this, the gender diversity within the editorial boards of education journals requires additional attention.

The inclusion of diverse perspectives within the composition of editorial boards is vital for the advancement of the journal. Firstly, membership on the editorial board of academic journals is commonly regarded as a symbol of influence, leadership, and prestige within the academic research community, thereby contributing to one’s scholarly standing and academic growth (Jagsi et al., 2020; Rivera et al., 2023). Editorial board members play a crucial role as significant gatekeepers, as they are responsible for making critical determinations regarding publication and shaping research trends within their respective fields. Additionally, scholarly evidence indicates that promoting equity in the scientific community not only fosters increased productivity and innovation, but also suggests that gender equity within editorial boards can enhance the review process (Wing et al., 2010). Moreover, the presence of diverse representation on these boards may have a profound influence on the development of future scientists. By promoting gender equity within journal editorial boards, academia can cultivate a more supportive and inclusive environment, ultimately leading to a more balanced and diversified scholarly community. When undergraduate students were randomly allocated to watch conference footage portraying either a predominantly male attendance or an equal representation of both genders, female students who observed the skewed attendance reported a diminished sense of belonging compared to their counterparts who witnessed balanced attendance. Conversely, male students’ sense of belonging remained unaffected by either scenario (Murphy et al., 2007). The absence of geographical diversity within an editorial committee presents considerable hazards to the caliber of academic publications. Geographical diversity encompasses a multitude of viewpoints stemming from distinct geographic regions, cultural contexts, and socio-economic environments. The absence of diverse geographical representation within an editorial committee can inadvertently sustain bias and ethnocentrism in the selection and evaluation of scholarly work (McKenzie et al., 2022). To address this issue, internationalizing editorial boards can help mitigate potential implicit biases. Furthermore, it is imperative to acknowledge that various regions frequently encounter unique challenges and prioritize different issues. In the absence of diverse representation, certain academic viewpoints may be disregarded or marginalized (Menges and Exum, 1983). This constraint can lead to an imbalanced portrayal of global matters, impeding the publication’s capacity to encompass the intricate facets of research and diminishing its pertinence to a wider readership. However, the presence of ample geographical diversity within the journal can enhance its appeal, as scientists who feel that their study will be subject to unfair judgment based on their nationality or location are more inclined to submit their manuscripts to journals with editors from their respective regions. Moreover, the lack of geographical diversity may hinder the committee’s ability to recognize and value the subtle cultural nuances embedded within academic works (Mammides et al., 2016). The comprehension of the cultural context is imperative for precise interpretation and assessment of research, particularly when addressing context-dependent subjects. Insufficient diversity can result in misinterpretations or oversights, thereby compromising the scholarly integrity of publications. Additionally, an editorial committee lacking geographical diversity may unintentionally perpetuate prevailing power imbalances within the academic domain. The underrepresentation or marginalization of specific regions or countries can perpetuate an inequitable allocation of influence and recognition, thereby hindering endeavors aimed at cultivating a genuinely global and inclusive academic dialogue.

Furthermore, it is evident that favorable advancements in education will inevitably yield beneficial outcomes in various other academic fields. The presence and influence of editorial board members play a crucial role in the dissemination of knowledge and the advancement of the discipline as a whole. Given that membership on editorial boards is typically determined by academic rank, achievements, and responsibilities (McMullen et al., 2022), the observed disparities in educational journals may account for variations in their influence. Consequently, the under-representation of certain groups, such as women and academics from developing countries, on editorial boards can have detrimental effects. Therefore, it is important to examine geographical and gender representation in order to assess their impact and contribution to editorial boards. The transformation of gender imparity into bias within research processes and outputs can be observed (Holdcroft, 2007). In fact, the introduction of diversity within a particular field enhances its overall efficiency. The amalgamation of individuals with varying life experiences not only fosters heightened intelligence and communication skills but also promotes a greater likelihood of undertaking proactive measures. This aspect holds particular significance within education domains, where the handling of intricate subjects is prevalent.

The under-representation of women on editorial boards can be attributed to a multitude of factors. Editors and members of editorial boards are typically researchers who possess excellent competence and an established reputation within their respective research specialties. They often exhibit a strong track record of published research, particularly as senior authors. Additionally, they typically possess significant experience in manuscript review. The lack of representation of females is likely attributable to various cultural and societal barriers that hinder equity and inclusion (Edmunds et al., 2016). It is imperative to exert diligent efforts in order to eradicate these barriers and enhance female representation in leader position and authorship. Academic platforms and organizations should collaborate in their efforts to cultivate the environment of inclusivity and equity within the academic publication. Additionally, they should actively endorse and support the equitable representation of women in editorial leadership roles.

Moreover, it is important to acknowledge that obvious geographical disparity was observed among editorial boards of education journals. The disproportionately low representation of editors from developing countries in education journals, and most editors came from English speaking countries, irrespective of gender considerations. A previous study has demonstrated that researchers hailing from high income countries are more highly valued compared to their counterparts from low/middle income countries (Sheikh et al., 2017). Additionally, there exists a dearth of authors from low/middle income countries in research publications pertaining to these regions, while authors from high-income countries, particularly male authors, exhibit a higher frequency of publications in this domain and are frequently cited (Memon et al., 2021). In contrast to their counterparts in developed nations, researchers in developing countries encounter numerous challenges, including limited financial resources, inadequate equipment, absence of mentorship, and linguistic disparities (Sumathipala et al., 2004; Lewison et al., 2016). Consequently, these obstacles significantly impede the ability of scholars from developing countries to attain the necessary academic qualifications for securing positions on editorial boards. Thus, urgent action is required to enhance the geographical diversity of education journal editorial boards.

The lack of representation of non-Anglophone countries may limit the contributions of researchers who are not proficient in English, which will further affect their probability of obtaining senior academic positions. The language discordance poses a significant impediment to achieving research equity. Nevertheless, there exist potential approaches to surmount this obstacle. One such approach involves translating articles into alternative languages subsequent to their initial publication, be it in written or audio format. In fact, numerous journals have been published in multiple languages, thereby mitigating the hindrances arising from language discordance (Shlobin et al., 2022). An additional approach involves implementing mechanisms to aid non-Anglophone authors in their English language writing or editing endeavors. Although certain publishers do provide translation services, their high costs often surpass the financial means of researchers from low-resource settings. However, it is encouraging to observe an increasing number of journals offering complimentary language editing services to authors (Amano et al., 2021). Consequently, we advocate for the implementation of additional policies of this nature to ameliorate the academic challenges stemming from language barriers. Moreover, it is crucial to acknowledge the beneficial impact of translation technologies and artificial intelligence on researchers hailing from non-Anglophone nations. Various valuable online resources, including reasonably dependable machine translations and platforms offering pronunciation solutions for multiple languages, are instrumental in overcoming the academic challenges arising from language barriers. Over time, advancements in machine translation technologies and collaborative endeavors to reshape academic conventions hold the potential to convert a monolingual scientific hub into a multilingual scientific network. Finally, we advocate for increased inclusivity in scientific endeavors. When deliberating on the selection of plenary speakers for conferences, extending invitations to join journal editorial boards, or engaging in the recruitment of new personnel, it is imperative to consciously strive for the inclusion of individuals who are not native English speakers. We firmly believe that offering genuine support to non-native speakers will enhance their ability to contribute valuable perspectives and ultimately maximize the benefits for academic development they bring.

The study’s limitation lied in its exclusive focus on the gender labels of men and women, neglecting the inclusion of gender-nonconforming, transgender, and gender nonbinary individuals. Additionally, contributions to the journal and academic influence of editorial board members were not assessed. At last, only the top five journals from each quartile were analyzed in our study, and focusing on high-level journals may lead to biased estimates of total diversity.

## Conclusion

Editorial boards of education journals exhibit reasonable gender diversity than other disciplines. However, there was a notable lack of representation of researchers associated with institutions from developing countries on the editorial boards of education journals. While maintaining sufficient gender diversity, it is imperative to enhance the geographical diversity in these journals, ensuring a more equitable number of positions to individuals from these underrepresented groups.

## Data availability statement

The raw data supporting the conclusions of this article will be made available by the authors, without undue reservation.

## Ethics statement

Ethical review and approval was not required for the study on human participants in accordance with the local legislation and institutional requirements. Written informed consent from the patients/participants or patients/participants’ legal guardian/next of kin was not required to participate in this study in accordance with the national legislation and the institutional requirements.

## Author contributions

YX: Writing – original draft. QX: Writing – review & editing.
